# The thyroid-heart axis-hormone dynamics and outcomes in cardiogenic shock following myocardial infarction

**DOI:** 10.1177/17511437261454174

**Published:** 2026-06-23

**Authors:** Priyanka Boettger, Jamschid Sedighi, Laura Pallmann, Patrick Kellner, Henning Lemm, Roland Prondzinsky, Thomas Karrasch, Karl Werdan, Michael Buerke

**Affiliations:** 1 Department of Internal Medicine I, Cardiology, Angiology and Intensive Care Medicine, Justus-Liebig-University, Giessen, Germany; 2 Department of Internal Medicine III (Cardiology, Angiology, Intensive Care Medicine), Martin-Luther-University Halle-Wittenberg, Halle (Saale), Germany; 3 Department of Anaesthesiology, Regio Klinikum Elmshorn, Germany; 4 Department of Internal Medicine II, Heart-Center-Südwestfalen, St. Marien-Hospital, Siegen, Germany; 5 Department of Internal Medicine III, Endocrinology, Justus-Liebig-University, Giessen, Germany

**Keywords:** cardiogenic shock, thyroid hormone, TSH, f T3, fT4, mortality, low T3 syndrome

## Abstract

**Background:**

Thyroid hormone alterations are common in critical illness and may reflect disease severity. Their prognostic significance in infarct-related cardiogenic shock remains incompletely defined.

**Methods:**

In this prospective cohort study from a cardiogenic shock registry, 41 patients with acute myocardial infarction complicated by cardiogenic shock underwent serial measurements of thyroid-stimulating hormone (TSH), free triiodothyronine (fT3), and free thyroxine (fT4) at baseline and 24, 48, 72, and 96 h after percutaneous coronary intervention. Associations between hormone trajectories and in-hospital mortality were assessed using longitudinal and small-sample–robust methods.

**Results:**

Thyroid trajectories diverged by outcome. Survivors maintained higher TSH concentrations over time, whereas non-survivors showed progressive suppression. fT3 declined in both groups early after presentation, with a steeper overall decline in non-survivors and outcome separation becoming most apparent later in the observation period. Lower fT3 was associated with greater illness severity and higher mortality risk. fT4 decreased over time in both groups without consistent between-group differences.

**Conclusions:**

In conclusion, dynamic alterations in thyroid hormone levels, particularly reductions in fT3, were associated with greater illness severity and adverse outcomes in cardiogenic shock. Serial thyroid function assessment may improve risk stratification in acute cardiovascular critical illness and warrants further evaluation in larger prospective studies.

## Introduction

Cardiogenic shock (CS) remains one of the most devastating complications of acute myocardial infarction, marked by impaired tissue perfusion, progressive multiorgan failure, and persistently high mortality despite advanced mechanical and pharmacologic interventions.^1^ The systemic nature of CS extends beyond hemodynamic collapse, involving a cascade of neurohumoral and endocrine responses that reflect both injury severity and the body’s attempt to restore homeostasis. Among these, alterations in thyroid hormone signaling, long recognized in critical illness^2,3^, have emerged as a potential biomarker and modulator of outcome, yet remain underexplored in cardiogenic shock.

### Role of T3 and T4 in shock physiology

Thyroid hormones, particularly triiodothyronine (T3) and thyroxine (T4), are essential regulators of metabolic rate, cardiovascular function, and cellular energy homeostasis. Synthesized in the thyroid gland through iodination of thyroglobulin, these hormones circulate largely bound to thyroxine-binding globulin (TBG), with T4 levels exceeding T3 concentrations by a factor of approximately 40. In peripheral tissues, T4 is converted by 5′-deiodinases into the biologically active T3, or into reverse T3 (rT3), an inactive metabolite, under tight regulation by the hypothalamic-pituitary-thyroid axis via thyrotropin-releasing hormone (TRH) and thyroid-stimulating hormone (TSH). T3 enhances intermediary metabolism, supports growth and tissue differentiation, and exerts powerful effects on the cardiovascular system.^4^

While low T3 syndrome has been described in various critical illnesses, including sepsis, acute myocardial infarction,^5^ myocarditis^6^ and heart failure^7^, its dynamic profile and prognostic implications in the context of cardiogenic shock following myocardial infarction remain underexplored. Triiodothyronine (T3) enhances cardiac output by lowering systemic vascular resistance, increasing β-adrenergic receptor density, and improving intracellular calcium cycling. In vascular smooth muscle, T3 induces relaxation and augments peripheral perfusion,^8^ mechanisms that may be critically relevant in the setting of circulatory collapse. Most prior studies have assessed thyroid hormone levels at a single time point and in heterogeneous populations.^9,10^ While Xue et al.^10^ showed baseline FT3/FT4 in cardiogenic shock complicating ST-segment elevation myocardial infarction, and Lymvaios et al.^11^ linked T3 to recovery after acute myocardial infarction, there remains a gap in understanding serial thyroid trajectories during recovery from cardiogenic shock. Our study adds value by focusing on serial hormone measurements in a narrowly defined cohort of cardiogenic shock patients undergoing acute revascularization. The observed association between early thyroid hormone decline and in-hospital outcomes may inform both risk stratification and future interventional strategies in this high-risk group.

### Thyroid response to critical illness

The response of the thyroid axis to acute illness, similar to the growth hormone, IGF-1 axis—is biphasic and context-dependent. Within hours of severe physiological stress such as trauma or myocardial infarction, serum T3 levels decline in proportion to disease severity, while T4 and TSH may transiently rise.^12,13^ Reduced activity of 5′-monodeiodinase enzymes, along with altered hormone binding and metabolism due to increased free fatty acids and bilirubin, contribute to this phenomenon.^9,14^ As the illness persists, T3 levels remain suppressed, nocturnal TSH secretion is blunted, and circulating rT3 increases: a constellation referred to as *low T3 syndrome* or *euthyroid sick syndrome*.^15^ These hormonal shifts are thought to represent an adaptive energy-conserving response mediated by inflammatory cytokines such as TNF-α, IL-1, and IL-6.^9,16^

In prolonged critical illness, both T3 and T4 levels decline further, often accompanied by a paradoxical suppression of TSH and a marked reduction in the pulsatile release of thyrotropin–a pattern suggestive of central dysregulation.^13,17^ Notably, this hormonal signature has been repeatedly observed in cardiac patients, including those with acute myocardial infarction, advanced heart failure, and following cardiac surgery, and has been independently associated with poor prognosis, including increased mortality.^2,18,19^

### Study rationale and objectives

Despite mounting evidence from cardiac and critical care cohorts, the prognostic significance of thyroid hormone changes in cardiogenic shock remains incompletely understood. In this prospective study, we aimed to determine:

1. Which alterations in thyroid hormone profiles are detectable in patients with CS?
2. Whether low T3 syndrome is associated with worse clinical outcomes than preserved thyroid function.
3. Whether changes in thyroid hormone levels carry prognostic value in the context of cardiogenic shock.

## Material and methods

### Study population and diagnostic criteria

Consecutive patients admitted to the academic hospital with myocardial infarction-related cardiogenic shock, defined by ST-elevation or non-ST-elevation myocardial infarction, were prospectively enrolled over a single-year period. Patients were excluded if they were referred for coronary artery bypass grafting during hospitalization or had incomplete thyroid hormone sampling; four patients met these criteria, leaving 41 patients in the final study cohort. The study was conducted in accordance with the Declaration of Helsinki and was approved by the local ethics committee; all participants provided written informed consent.

Cardiogenic shock was diagnosed at the time of initial coronary angiography on the basis of the following criteria:

- Persistent hypotension, defined as systolic blood pressure (SBP) < 90 mmHg for >30 min or the need for vasopressor therapy/mechanical support to maintain SBP > 90 mmHg;
- Clinical signs of systemic hypoperfusion (e.g. oliguria/anuria < 30 mL/h, cold/cyanotic extremities, or signs of cerebral hypoperfusion);
- Indexed cardiac output < 2.2 L/min/m^2^;
- Pulmonary capillary wedge pressure > 15 mmHg.

Patients referred for cardiothoracic surgery during hospitalization were excluded due to the non-comparability of postoperative endocrine responses. None of the patients had a known history of hypo- or hyperthyroidism, and none were receiving thyroid-related medication. All patients underwent coronary angiography with iodinated contrast agents.

### Data collection and sampling schedule

Clinical data were collected at predefined time points: at admission (“initial”), pre- and post-percutaneous coronary intervention (PCI), 6 h after catheterization, daily from day 1 to 7, and on days 14 and 28. Blood samples for hormonal and biochemical analyses were drawn at baseline and at 24-, 48-, and 72-h post-PCI. Intensive care scoring systems, Acute Physiology and Chronic Health Evaluation II and III (APACHE II and III), and Sequential Organ Failure Assessment (SOFA) score, were assessed at baseline and again at 24-, 48-, 72-, and 96-h post-PCI. Routine laboratory tests were conducted between 7:00–8:00 a.m. on days 0–7, 14, and 28, and included:

- Electrolytes, complete blood count, and coagulation profile
- Cardiac biomarkers (e.g. myocardial enzymes)
- Organ function parameters (renal, hepatic, pancreatic)
- Metabolic markers (e.g. serum proteins, lipid profile, glucose)
- Inflammatory markers

### Hormone sample processing

Serum levels of thyroid-stimulating hormone (TSH), free triiodothyronine (fT_3_), and free thyroxine (fT_4_) were measured in fasting blood samples drawn between 7:00–08:00 a.m. at baseline and during follow-up. Additionally, 10 mL of venous blood was collected into two Sarstedt serum tubes, centrifuged, and stored at –80°C until batch analysis. TSH was measured using a third-generation high-sensitivity immunoassay (limit of detection < 0.01 mU/L; Roche Elecsys), and fT_3_/fT_4_ via automated immunoassays (Beckman Coulter) in the Endocrinology Laboratory.

Based on thresholds established in the literature for patients with cardiovascular disease a cut-off of <3.1 pmol/L was used to define low T3 syndrome.^15,20^ Laboratory reference ranges were 0.35–3.50 mU/L for TSH, 3.1–6.8 pmol/L for fT3, and 7.7–14.2 pmol/L for fT4. The literature-based threshold used to define low T3 syndrome (<3.1 pmol/L) differs from the lower limit of the laboratory reference range and was selected to reflect prognostic associations reported in prior studies.^9^ Exposure to vasoactive agents, inotropes, and mechanical circulatory support during the first 72 h was recorded to characterize clinical management and is summarized in Supplemental Table S1.

### Statistics

Analyses were conducted using IBM SPSS Statistics version 27 (IBM Corp., Armonk, NY, USA) and R version 4.4.1 (R Foundation for Statistical Computing, Vienna, Austria). Continuous variables are reported as mean ± SD or median with interquartile range, depending on distribution (Shapiro–Wilk test). Group comparisons used independent t-tests or Mann–Whitney *U* tests for continuous variables and χ^2^ or Fisher’s exact tests for categorical variables. Correlations with clinical parameters were assessed by Pearson’s or Spearman’s coefficients.

Temporal changes in thyroid hormones (TSH, fT3, fT4) were analyzed by linear mixed-effects models with group-by-time interaction; area-under-the-curve (AUC) values for 96-h trajectories were computed using the trapezoidal rule. To address potential small-sample bias, we applied Firth-penalized logistic regression for mortality, permutation testing (10,000 iterations) for group differences, and bootstrap resampling (10,000 iterations) for bias-corrected confidence intervals and optimism-corrected AUCs.^21^ Bayesian logistic regression with weakly informative priors was used as a sensitivity analysis to estimate posterior odds ratios and credible intervals.^22^ Effect sizes are reported as Cohen’s *d* where applicable.^23^ A two-sided *p* < 0.05 was considered statistically significant. Vasoactive therapies and mechanical circulatory support were considered as potential covariates but were not included in multivariable models due to collinearity with shock severity and limited sample size; instead, they were examined descriptively to contextualize endocrine trajectories.

#### Power and multiplicity

Owing to the limited cohort size (*n* = 41; 18 deaths), analyses were considered exploratory and aimed at estimation and pattern detection rather than confirmatory hypothesis testing. The number of covariates in mortality models was restricted to minimize overfitting given the available number of events. Because thyroid hormones were assessed at multiple time points and across outcomes, we interpreted timepoint-specific *p*-values cautiously and focused on effect sizes, confidence/credible intervals, and consistency across complementary modeling approaches. Accordingly, effect estimates should be interpreted as exploratory measures of association rather than precise estimates of effect.

## Results

### Clinical characteristics of the patients

Of 45 consecutively enrolled patients with myocardial infarction, related cardiogenic shock, four were excluded from the analysis, three because of referral for coronary artery bypass grafting and one because of incomplete thyroid hormone sampling, leaving 41 patients in the final study cohort. The average age at admission was 67.5 ± 10.9 years (median: 70; range: 43–85). Female patients were 3.5 years older on average than males (69.7 ± 10.1 years vs 66.2 ± 9.6 years; *p* = 0.21; 95% CI: −9.2 to 2.1). Survivors were significantly younger than non-survivors (64.9 ± 9.8 years vs 70.8 ± 11.4 years; *p* = 0.04; 95% CI: −11.6 to −0.2), with a moderate effect size (Cohen’s *d* = 0.56). Nearly half (48.8%) were aged 65–75 years (Table 1).

**Table 1.** Baseline characteristics of the study population values are presented as mean ± standard deviation or number (percentage).

Variable	All patients (*n* = 41)	Survivors (*n* = 23)	Non-survivors (*n* = 18)	Male (*n* = 26)	Female (*n* = 15)	Age < 70 years (*n* = 18)	Age ⩾ 70 years (*n* = 23)	*p*-Value (survivors vs non-survivors)
Age (years)	67.5 ± 10.9	64.9 ± 9.8	70.8 ± 11.4	66.2 ± 9.6	69.7 ± 10.1	59.2 ± 8.5	76.2 ± 9.7	**0.04**
Weight (kg)	87.1 ± 13.6	88.7 ± 12.4	84.9 ± 15.2	84.1 ± 14.1	88.8 ± 12.2	91.4 ± 11.7	82.6 ± 14.6	0.21
Height (cm)	171.2 ± 8.2	174.3 ± 7.4	167.3 ± 8.6	176.0 ± 6.8	162.9 ± 7.2	177.0 ± 6.3	165.2 ± 7.8	0.06
Prior MI, *n* (%)	10 (24)	7 (30)	3 (17)	3 (12)	7 (47)	4 (22)	6 (26)	0.28
Prior HF, *n* (%)	17 (41)	9 (39)	8 (44)	5 (19)	12 (80)	5 (28)	12 (52)	0.53
BMI (kg/m^2^)	29.9 ± 6.3	29.3 ± 6.1	30.4 ± 6.6	28.7 ± 5.9	31.5 ± 6.7	29.4 ± 5.8	30.2 ± 6.5	0.28
Hypertension, *n* (%)	26 (63)	17 (74)	9 (50)	15 (58)	11 (73)	13 (72)	13 (57)	0.10
Type 2 DM, n (%)	24 (59)	14 (61)	10 (56)	12 (46)	12 (80)	10 (56)	14 (61)	0.71
Hyperlipidemia, *n* (%)	8 (20)	6 (26)	2 (11)	2 (8)	6 (40)	5 (28)	3 (13)	0.20
Current smoking, *n* (%)	9 (22)	6 (26)	3 (17)	2 (8)	7 (47)	7 (39)	2 (9)	0.38

AMI: acute myocardial infarction; BMI: body mass index; DM: diabetes mellitus; HF: heart failure; MI: myocardial infarction.

Bold values indicate statistical significance (*p* < 0.05).

The cohort showed a high cardiovascular risk burden: each patient had an average of 3.5 risk factors; 87.8% had ⩾2. Risk factors included arterial hypertension, diabetes, dyslipidaemia, smoking, obesity, and family history of cardiovascular disease. Anthropometric data showed a mean weight of 87.1 kg (range: 60–125), height 171.2 cm (range: 145–190), and body mass index (BMI) of 29.9 ± 6.3 kg/m^2^ (range: 20.7–46.9). According to the World Health Organization (WHO) categories, most patients were overweight (BMI 25–30) or obese (BMI > 30). Female patients had a higher mean BMI than males (31.5 ± 6.7 kg/m^2^ vs 28.7 ± 5.9 kg/m^2^; *p* = 0.08; 95% CI: −0.43 to 5.97); 60% of women were obese, compared to 30% of men. Patients with BMI < 25 had an average of 2.0 cardiovascular risk factors, versus 4.0 among those with BMI > 25 (*p* < 0.001). Nearly one-third of overweight/obese patients reported ⩾5 risk factors. No correlation was found between BMI and age (*r* = 0.11, *p* = 0.48), suggesting body weight was not age dependent. In-hospital survival was lower in patients aged ⩾ 70 years (43.5%) compared to <70 years (72.2%), corresponding to a relative risk of 0.60 (95% CI: 0.34–1.04; χ^2^ = 3.42; *p* = 0.064).

### Routine laboratory parameters, organ dysfunction, and their association with thyroid hormones

On admission, laboratory investigations reflected the systemic severity of cardiogenic shock. Electrolyte disturbances were frequent, with hyponatremia in 32% and hypokalemia in 18%. The complete blood count revealed moderate leucocytosis (mean 12.4 ± 3.1 × 10^9^/L), consistent with systemic inflammation, while thrombocytopenia (<150 × 10^9^/L) was present in 22%. Coagulation abnormalities were observed in 27%, with elevated INR (>1.5) or reduced fibrinogen levels. Cardiac biomarkers were markedly elevated (troponin I mean 23.0 ± 5.1 μg/L; CK and CK-MB followed a similar pattern), reflecting myocardial necrosis, without significant differences between survivors and non-survivors (troponin I: 20.8 ± 5.8 μg/L vs 26.1 ± 9.1 μg/L; *p* = 0.42; 95% CI, −7.9 to 19.5). Organ dysfunction was evident across multiple systems. Acute kidney injury (AKI) was present in 41% at admission (mean creatinine 1.9 ± 0.6 mg/dL). Hepatic involvement was indicated by elevated transaminases (AST mean 92 U/L; ALT mean 78 U/L) and hyperbilirubinemia in 24%, while pancreatic enzymes were mildly elevated in 15%. Metabolic derangements included hypoalbuminemia (mean 27.8 ± 4.9 g/L) and dyslipidaemia with low HDL cholesterol (0.9 ± 0.2 mmol/L). Admission hyperglycemia (>180 mg/dL) occurred in 44%, reflecting stress metabolism.

Inflammatory markers were universally raised, with C-reactive protein (CRP) averaging 156 ± 54 mg/L, significantly higher in non-survivors than survivors (190 ± 49 mg/L vs 128 ± 42 mg/L; *p* = 0.008; 95% CI, 16.7–104.9). Importantly, CRP correlated inversely with fT3 (Spearman *r* = −0.44; *p* = 0.01). Lower fT3 showed moderate inverse correlations with markers of renal dysfunction (creatinine: *r* = −0.38; *p* = 0.02), hepatic impairment (bilirubin: *r* = −0.41; *p* = 0.02), and hypoalbuminemia (*r* = 0.35; *p* = 0.03). These associations were weaker for fT4 (SOFA correlation *r* = −0.28; *p* = 0.07) and absent for TSH (*r* = −0.12; *p* = 0.41). Lower thyroid hormone levels, particularly fT3, were observed in parallel with markers of systemic inflammation and organ dysfunction, reflecting global disease severity in cardiogenic shock.

### Hemodynamic profile and initial management

At admission, patients presented with advanced cardiogenic shock, with a mean systolic blood pressure (SBP) of 104 mmHg and heart rate of 94 beats per minute. Non-survivors had significantly lower SBP compared with survivors (88.5 mmHg vs 116.6 mmHg; *p* = 0.001; 95% CI, −42.3 to −11.4). By the end of the first hospital day, SBP values converged (100.1 mmHg vs 108.1 mmHg), although a trend toward greater hemodynamic stability in survivors persisted (*p* = 0.06). The mean baseline cardiac index was 1.96 ± 0.20 L/min/m^2^, improving to 2.57 ± 0.30 L/min/m^2^ on day 1 following revascularization and stabilization.

Almost all patients (97.6%) underwent urgent cardiac catheterization, with stent implantation in two-thirds, and more than 70% receiving intervention within 2 h of admission.

The severity of circulatory failure was underscored by the frequent need for advanced support: >75% required mechanical circulatory support and >80% received combined norepinephrine and dobutamine therapy, with epinephrine used in 22%. Levosimendan was administered in 44% as adjunctive inotropic support. Antithrombotic treatment was universal, with aspirin and unfractionated heparin given at admission; additional therapies included glycoprotein IIb/IIIa inhibitors, ACE inhibitors, nitrates, β-blockers, and amiodarone. Details on vasoactive therapy and mechanical circulatory support are provided in Supplemental Table S1.

### Severity scores and their association with thyroid hormones

At baseline, the cohort exhibited marked illness severity, with a median APACHE II score of 24 (IQR 20–29), APACHE III of 74 (IQR 63–92), and SOFA of 10 (IQR 8–12). Non-survivors presented with significantly higher values across all scores compared with survivors (APACHE II: 28 vs 22, *p* = 0.006; APACHE III: 88 vs 66, *p* = 0.003; SOFA: 12 vs 8, *p* < 0.001), and their scores remained persistently elevated over the first 96 h. In survivors, SOFA declined steadily from a median of 8 at admission to 3 at 96 h, whereas in non-survivors it remained stable or increased (12 to 14; group × time interaction *p* < 0.001).

**Figure 1.**
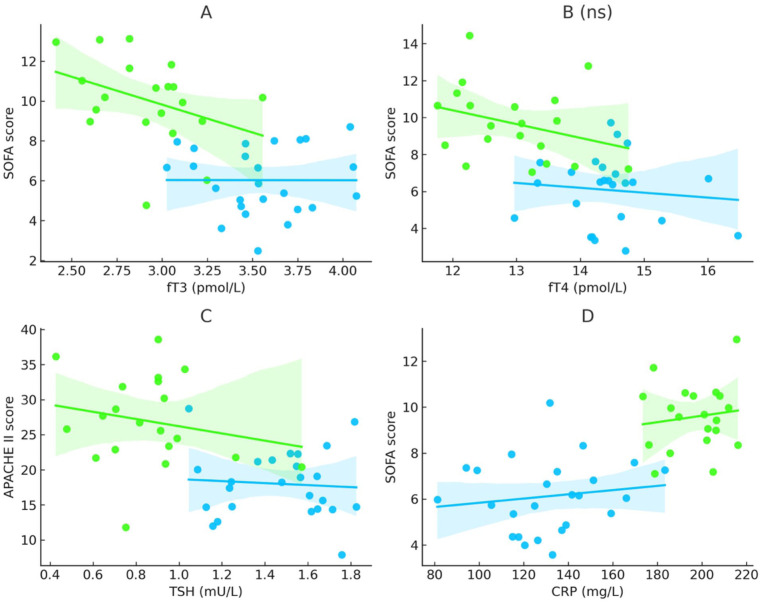
Associations of thyroid hormones and inflammation with illness severity in cardiogenic shock. Panel A illustrates the inverse association between free triiodothyronine (fT3) and SOFA scores, with non-survivors showing lower fT3 values and higher illness severity (Spearman *r* = −0.46, *p* = 0.004). Panel B depicts the relationship between free thyroxine (fT4) and SOFA scores, showing a similar directional trend in both groups without reaching statistical significance (*r* = −0.28, *p* = 0.07). Panel C shows thyroid-stimulating hormone (TSH) in relation to APACHE II scores, demonstrating lower TSH levels in non-survivors and a significant inverse association (*r* = −0.39, *p* = 0.014). Panel D highlights the association between systemic inflammation and illness severity, with higher C-reactive protein (CRP) levels correlating with higher SOFA scores and being more pronounced in non-survivors (190 ± 49 mg/L vs 128 ± 42 mg/L; *p* = 0.008). Collectively, these findings indicate that suppression of thyroid hormones—particularly fT3 and TSH—parallels disease severity, while elevated CRP reflects the inflammatory burden associated with adverse outcomes in cardiogenic shock.

Severity scores correlated closely with thyroid hormone dynamics. Lower fT3 was strongly associated with higher SOFA (*r* = −0.46, *p* = 0.004) and APACHE II (*r* = −0.39, *p* = 0.014). Patients with low T3 syndrome (<3.1 pmol/L) had significantly higher SOFA (13 vs 9, *p* = 0.002) and APACHE II (29 vs 23, *p* = 0.009) scores. fT4 showed only a weak, non-significant inverse relationship with SOFA (*r* = −0.28, *p* = 0.07), whereas TSH correlated inversely with APACHE III (*r* = −0.31, *p* = 0.049). In multivariable analysis adjusting for age, sex, blood pressure, and lactate, fT3 remained an independent predictor of SOFA at 72 h (β = −0.38 points per 0.1 pmol/L increase; 95% CI, −0.64 to −0.12; *p* = 0.006). Suppressed thyroid hormone levels, particularly fT3, were observed in parallel with greater organ dysfunction severity in patients with cardiogenic shock (Figure 1).

### Analysis of the thyrotropic axis

**Figure 2.**
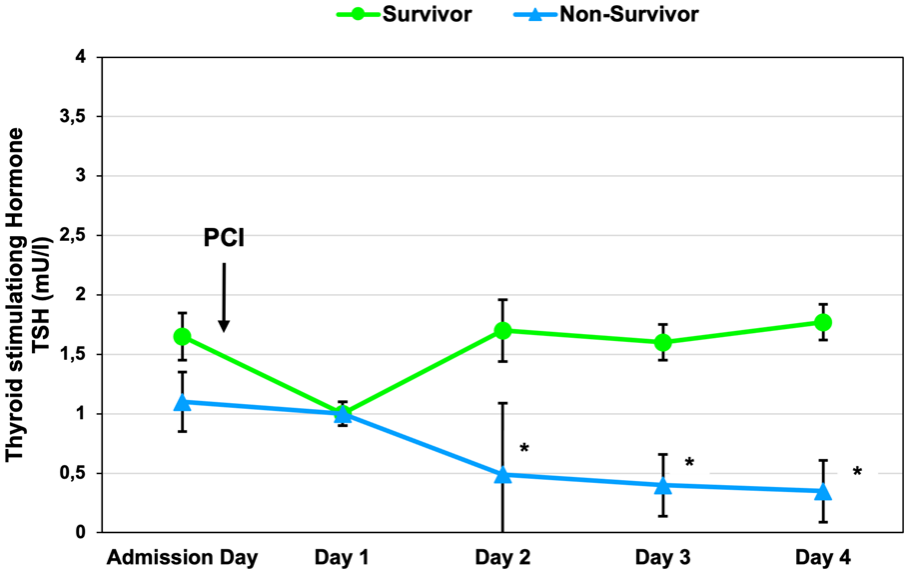
Time course of thyroid-stimulating hormone (TSH) concentrations in survivors and non-survivors with cardiogenic shock. TSH levels (mean ± SEM) were measured at admission and at 24, 48, 72, and 96 h after hospital admission and revascularization. Survivors are shown in blue and non-survivors in green. While baseline TSH concentrations were comparable between groups, non-survivors exhibited a progressive decline over time, resulting in persistently lower TSH levels compared with survivors from 48 h onward. Cumulative TSH exposure over 96 h was lower in non-survivors (AUC_0_–_96_ = 51.8) than in survivors (AUC_0_–_96_ = 84.6), consistent with sustained suppression of the hypothalamic–pituitary axis. Dashed horizontal lines indicate the laboratory reference range (lower normal limit [LNL] = 0.4 mU/L; upper normal limit [UNL] = 3.5 mU/L). Asterisks (*) denote time points at which TSH levels differed significantly between survivors and non-survivors (*p* < 0.05).

Serial profiling of TSH, fT3, and fT4 revealed early and prognostically relevant hormonal divergence in cardiogenic shock. At baseline, mean TSH was 1.42 ± 0.22 mU/L (median, 0.88; range, 0.05–6.10), with most values within the reference range (0.35–3.50; Figure 2). TSH concentrations were broadly comparable between women and men, whereas younger patients (<70 years) tended to exhibit higher levels throughout follow-up (Table 2). Baseline TSH levels were numerically higher in survivors than in non-survivors (1.65 mU/L vs 1.10 mU/L). At day 1, TSH levels decreased in both groups and were similar between survivors and non-survivors. Thereafter, trajectories diverged over time: survivors showed recovery of TSH levels, whereas non-survivors exhibited a sustained decline, with statistically significant differences observed from day 2 onward (Figure 2).

**Table 2.** Mean TSH concentrations by sex and age group values are presented as mean ± standard error.

Timepoint	Female (mU/L)	Male (mU/L)	<70 years (mU/L)	⩾70 years (mU/L)
Initial	1.39 ± 0.40	1.44 ± 0.26	1.93 ± 0.44	1.02 ± 0.14
24 h	0.99 ± 0.15	0.96 ± 0.29	1.19 ± 0.40	0.81 ± 0.11
48 h	1.18 ± 0.45	1.43 ± 0.56	1.66 ± 0.74	1.08 ± 0.36
72 h	0.88 ± 0.22	1.12 ± 0.42	1.06 ± 0.49	1.00 ± 0.27
96 h	1.36 ± 0.42	1.26 ± 0.40	1.33 ± 0.40	1.26 ± 0.42

TSH: thyroid-stimulating hormone.

**Figure 3.**
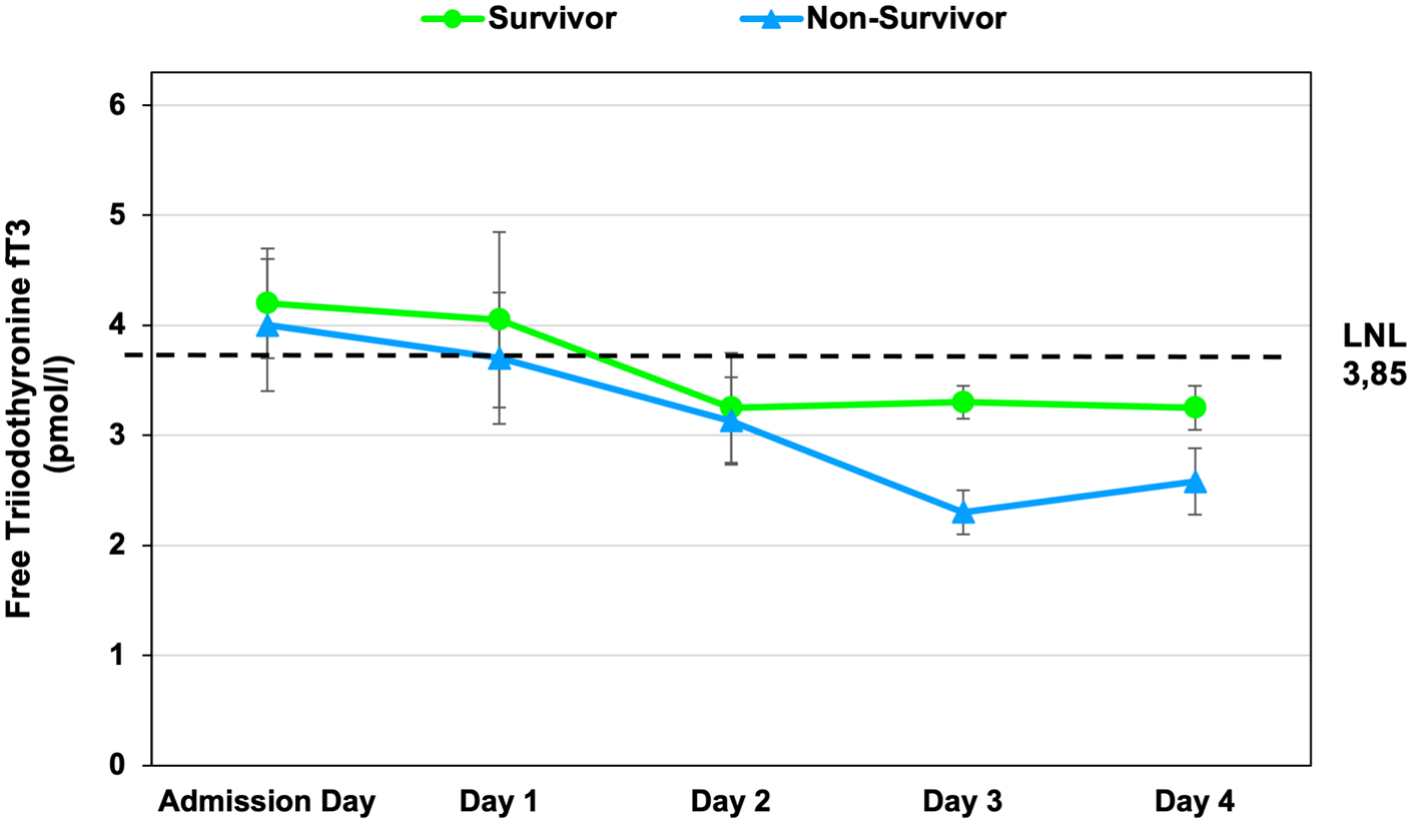
Time course of free triiodothyronine (fT3) concentrations in survivors and non-survivors with cardiogenic shock. Free triiodothyronine (fT3) levels (mean ± SEM) were measured at admission and at 24, 48, 72, and 96 h after hospital admission and revascularization. Both groups showed an early decline in fT3 concentrations. Over time, trajectories diverged, with lower fT3 levels in non-survivors at later time points. The dashed horizontal line indicates the lower normal limit of the laboratory reference range (LNL = 3.85 pmol/L).

Baseline fT3 averaged 4.12 ± 0.22 pmol/L (median, 3.95; range, 1.72–8.58). Age-related differences in fT3 concentrations were more pronounced than sex-related differences, with consistently higher values among patients aged <70 years throughout follow-up (Table 3). Concentrations declined in all patients, but the slope differed by outcome. Survivors showed a modest decrease (3.99–3.29 pmol/L; Δ = −0.70; *p* = 0.073; 95% CI, −1.49 to 0.05), whereas non-survivors had a steeper fall (4.28–3.19 pmol/L; Δ = −1.19; *p* = 0.011; 95% CI, −2.12 to −0.31; Figure 3).

**Table 3.** Mean fT3 concentrations by sex and age group values are presented as mean ± standard deviation.

Timepoint	Female (pmol/L)	Male (pmol/L)	<70 years (pmol/L)	⩾70 years (pmol/L)
Admission	4.6 ± 1.5	4.2 ± 0.8	5.1 ± 1.2	3.6 ± 0.9
24 h	4.0 ± 1.2	3.5 ± 0.7	4.4 ± 1.0	3.1 ± 0.8
48 h	3.6 ± 1.1	3.4 ± 0.6	4.2 ± 0.9	2.9 ± 0.7
72 h	3.1 ± 0.9	3.3 ± 0.6	3.9 ± 0.8	2.6 ± 0.6

fT3: free triiodothyronine.

**Figure 4.**
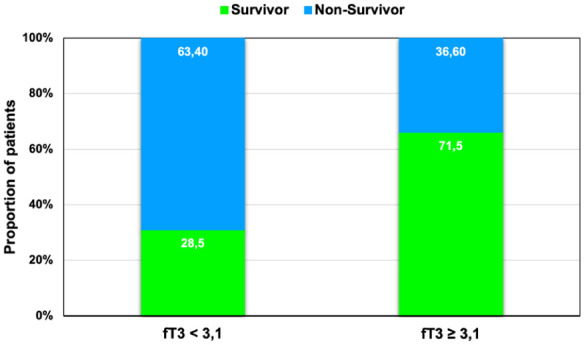
Distribution of survival status according to fT3 category. Patients were classified as having low T3 syndrome if their serum free triiodothyronine (fT3) concentration was <3.1 pmol/L at any point during the first 72 h. Bars represent the proportion of survivors and non-survivors within each fT3 category (<3.1 pmol/L vs ⩾3.1 pmol/L). Low T3 syndrome was observed in 29.4% of non-survivors (5/17) and 8.7% of survivors (2/23), corresponding to a relative risk of 3.37 (95% CI, 0.76–15.0; *p* = 0.11). This difference did not reach statistical significance.

Between-group differences in fT3 decline were significant (*p* = 0.045). Low T3 syndrome was observed in 29.4% of non-survivors compared with 8.7% of survivors; the distribution of survival status according to fT3 category is shown in Figure 4, corresponding to a relative risk of death of 3.37 (95% CI, 0.76–15.0; *p* = 0.11). This finding did not reach statistical significance.

**Figure 5.**
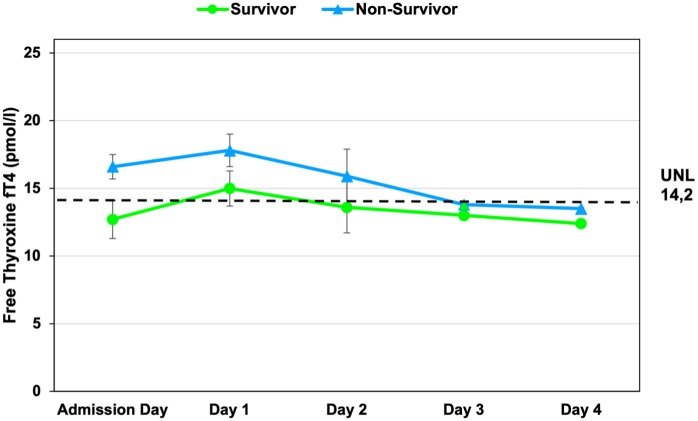
Time course of free thyroxine (fT4) concentrations in survivors and non-survivors with cardiogenic shock. Free thyroxine (fT4) levels (mean ± SEM) were measured at admission and at 24, 48, 72, and 96 h after hospital admission and revascularization. Both groups exhibited a gradual decline in fT4 concentrations over time. Non-survivors showed numerically higher fT4 levels at early time points, with convergence of values during follow-up. The dashed horizontal line denotes the upper normal limit of the laboratory reference range (UNL = 14.2 pmol/L).

Mean baseline fT4 was 15.05 ± 0.75 pmol/L (median, 13.95; range, 9.30–30.60), modestly above the reference range (7.7–14.2). Values declined gradually in both groups, falling below the lower limit of normal by 72 h. Between-group differences were not significant (*p* > 0.10; Figure 5). Broad interindividual variability, as summarized in Table 4, likely obscured subtle subgroup effects. Early suppression of fT3 and lower TSH levels were observed in non-survivors compared with survivors (Figures 2 and 3).

**Table 4.** Mean fT4 concentrations by sex and age group values are presented as mean ± standard deviation.

Timepoint	Female (pmol/L)	Male (pmol/L)	<70 years (pmol/L)	⩾70 years (pmol/L)
Admission	17.75 ± 2.35	14.61 ± 0.85	14.90 ± 1.93	16.43 ± 1.05
24 h	17.65 ± 2.26	16.20 ± 1.08	16.26 ± 1.84	17.11 ± 1.19
48 h	17.30 ± 3.13	14.92 ± 0.93	15.28 ± 2.88	16.30 ± 0.78
72 h	16.08 ± 3.32	13.37 ± 1.05	14.28 ± 2.87	14.55 ± 0.79
96 h	15.73 ± 2.73	12.69 ± 0.82	14.00 ± 2.40	13.74 ± 0.57

fT4: free thyroxine.

### 30-Day outcomes

**Figure 6.**
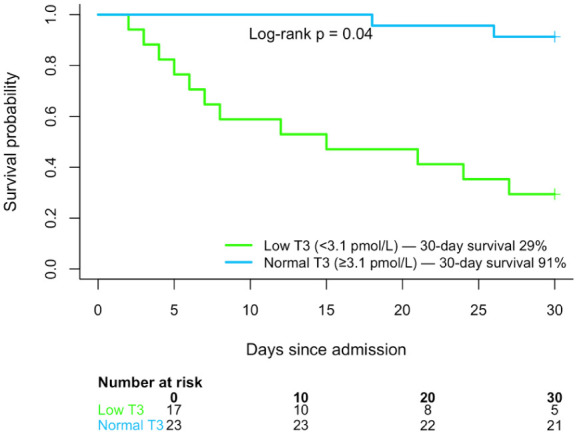
Kaplan–Meier survival according to baseline free triiodothyronine (fT3) status. Survival probability was stratified by baseline fT3 concentration, with low T3 syndrome defined as fT3 <3.1 pmol/L at admission. Patients with low baseline fT3 showed reduced survival compared with those with preserved fT3 levels (log-rank *p* = 0.04). By 30 days, survival was approximately 30% in the low T3 group versus >90% in the normal T3 group. Numbers at risk are shown below the plot.

Follow-up through 30 days was complete in all 41 patients. In addition to the 18 in-hospital deaths (43.9%), two deaths occurred after hospital discharge, resulting in a cumulative 30-day mortality of 48.8% (20 of 41 patients). Kaplan–Meier analysis indicated that deaths occurred predominantly in the early phase after admission, with a higher density of events within the first 10–14 days, followed by a relative attenuation in event rates thereafter (Figure 6).

Among 30-day survivors (*n* = 21), acute kidney injury occurred in 10 patients (47.6%), and renal replacement therapy was required in six patients (28.6%). Mechanical circulatory support had been used in 17 survivors (81.0%), with successful weaning in 14 patients (82.4%).

Patients with low T3 syndrome showed significantly reduced 30-day survival compared with those with preserved fT3 levels (29% vs 91%; log-rank *p* = 0.04; Figure 6), indicating an association between early fT3 suppression and adverse short-term outcomes. In a Cox proportional-hazards model adjusted for age, systolic blood pressure at admission, and serum lactate, low T3 syndrome was associated with an increased risk of death through day 30 (hazard ratio, 2.6; 95% CI, 1.1–6.4; *p* = 0.028).

### One-year follow-up

Between 30 days and 1 year, one additional death occurred, resulting in a cumulative 1-year mortality of 51.2% (21 of 41 patients). Vital status at 1 year was available for 40 of 41 patients, with one patient lost to follow-up. Among patients who survived beyond 30 days (*n* = 21), 1-year survival was 95.0% (19 of 20 patients with available follow-up data).

In a Cox proportional-hazards model adjusted for age, systolic blood pressure at admission, and serum lactate, low T3 syndrome was associated with increased 1-year mortality (hazard ratio, 2.1; 95% CI, 1.0–4.5).

### Small-sample-robust analyses

To address potential small-sample bias, we applied complementary statistical approaches. In Firth-penalized logistic regression, lower fT3 remained independently associated with in-hospital mortality after adjustment for age, systolic blood pressure at admission, and lactate (adjusted OR per 0.1 pmol/L decrease, 1.12; 95% CI, 1.03–1.25). Permutation testing (10,000 iterations) confirmed a greater decline in fT3 from admission to 72 h in non-survivors than in survivors (*p* = 0.041), alongside significant differences in SOFA trajectories (*p* < 0.001). Bootstrap resampling yielded bias-corrected confidence intervals consistent with the primary estimates and indicated improved discrimination when combining SOFA at 24 h with fT3 (optimism-corrected AUC, 0.84 vs 0.72).

Bayesian logistic regression produced concordant results (posterior OR per 0.1 pmol/L decrease, 1.11; 95% credible interval, 1.02–1.24; posterior probability = 0.97). In Kaplan–Meier analysis, low T3 syndrome was associated with reduced survival (log-rank *p* = 0.04), and in adjusted Cox regression with increased mortality risk (HR, 2.8; 95% CI, 1.1–7.0; *p* = 0.03). Across methods, lower fT3 remained consistently associated with mortality, although effect estimates were imprecise owing to the limited sample size.

## Discussion

In this prospective study of patients with cardiogenic shock, we demonstrate that thyroid hormone dynamics,particularly changes in fT3, fT4, carry prognostic information and are associated with adverse outcomes. While initial levels of TSH, fT3 and fT4,were within the reference range in most patients, temporal divergence emerged during the early clinical course. Both survivors and non-survivors showed an early decline in free triiodothyronine; however, prognostically relevant separation between groups became evident from approximately 72 h onward, with non-survivors exhibiting sustained suppression of fT3 alongside persistently lower TSH levels. The association between these hormonal trajectories and mortality was consistent across age and sex subgroups, underscoring the clinical relevance of thyroid axis dysregulation in the setting of profound circulatory failure.

These findings are in line with previous observations that non-thyroidal illness syndrome, especially low fT3 in the setting of preserved TSH, is common in critical illness and correlates with severity and outcome.^13,24^ The strikingly higher prevalence of low triiodothyronine syndrome in non-survivors, more than threefold compared to survivors, supports its role not merely as an epiphenomenon, but as a pathophysiological marker of disease progression. Prior studies in patients with acute respiratory distress syndrome have similarly shown that the extent of early triiodothyronine decline tracks closely with mortality risk.^25^ In our cohort, both groups exhibited an early decline in free triiodothyronine, whereas prognostically relevant divergence in thyroid hormone trajectories emerged later in the observation period, around 72 h, suggesting a dynamic endocrine response during early intensive care. In this context, the temporal distribution of mortality observed in the present cohort is consistent with contemporary outcome data in cardiogenic shock. Large registry studies and randomized trials report 30-day mortality rates of approximately 30%–40% and 1-year mortality approaching 40%–50%, despite guideline-directed management and early revascularization, with the majority of deaths occurring within the first days to weeks after shock onset.^26–28^ In our cohort, mortality was similarly concentrated in the early phase, with a 30-day mortality of 48.8%, and 85% of fatal events occurring within the first 10 days. Beyond this high-risk period, only one additional death occurred between 30 days and 1 year, indicating limited late attrition among early survivors. Notably, the association between low T3 syndrome and mortality was driven predominantly by early events, as reflected by markedly reduced 30-day survival in patients with suppressed fT3. This pattern does not indicate a sustained independent mortality risk beyond the acute phase, as survival among 30-day survivors remained largely stable during extended follow-up.

Interpretation of thyroid hormone trajectories in cardiogenic shock must also account for the fact that mechanical circulatory support modalities and vasoactive therapies primarily reflect overall shock severity and indication, rather than representing independent biological modifiers, and were therefore reported descriptively. Norepinephrine exposure should be interpreted as a marker of circulatory failure and organ dysfunction, while amiodarone is a recognized modulator of peripheral T4-to-T3 conversion and may have influenced observed hormone patterns.

In addition, differences in vasoactive and inotropic support between groups provide important clinical context for the observed endocrine patterns. Non-survivors required higher peak norepinephrine doses and longer durations of support, along with more frequent use of epinephrine and advanced mechanical circulatory support, consistent with more severe circulatory failure and greater hemodynamic instability.^29^ In contrast, levosimendan was used more frequently in survivors, which may reflect preserved myocardial contractile reserve or differences in treatment selection.^30,31^ These patterns likely represent underlying disease severity and confounding by indication rather than independent treatment effects and should be interpreted accordingly (Supplemental Table S1).

Beyond pharmacological effects, inflammation-related alterations in iodothyronine deiodinase activity, particularly induction of type 3 deiodinase (D3), provide a mechanistic link between immune activation, myocardial dysfunction, and reduced bioactive T3 levels.^32^ Increased D3 expression has been described in leukocytes during sepsis and in cardiomyocytes in dilated cardiomyopathy,^33,34^ leading to systemic and local inactivation of thyroid hormones despite preserved circulating levels.^35,36^ These mechanisms offer a plausible biological framework for the observed thyroid hormone patterns in cardiogenic shock, without implying causality in the present observational cohort.

**Figure 7.**
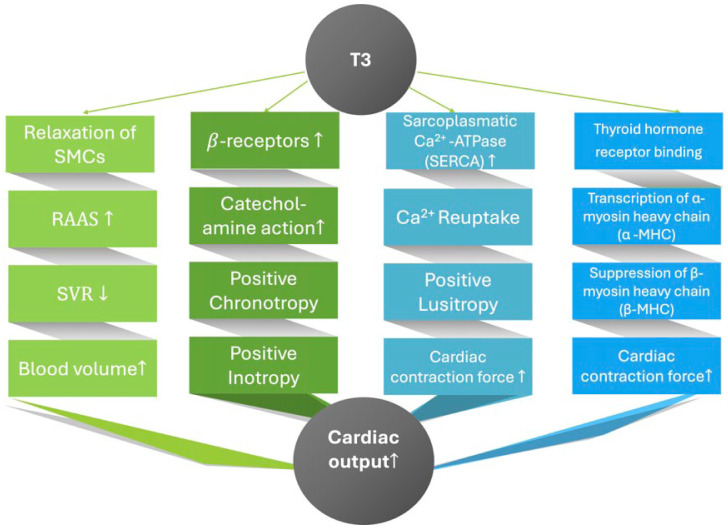
Modulation of hemodynamic pathways by triiodothyronine (T3) in acute cardiogenic shock. T3 enhances cardiac output (CO↑) through multiple direct and indirect mechanisms. These include: (1) vascular smooth muscle cell (SMC) relaxation with a reduction in systemic vascular resistance (SVR↓), accompanied by neurohumoral activation of the renin–angiotensin–aldosterone system (RAAS) contributing to increased circulating blood volume; (2) upregulation of β-adrenergic receptor expression and enhanced catecholamine sensitivity, resulting in positive chronotropic and inotropic effects; (3) improved intracellular calcium handling via increased sarcoplasmic Ca^2^⁺-ATPase (SERCA) activity, promoting efficient calcium reuptake and positive lusitropy; and (4) transcriptional regulation of contractile proteins, including increased α-myosin heavy chain and suppression of β-myosin heavy chain expression, leading to enhanced myocardial contractile force. Collectively, these mechanisms may contribute to improved cardiac performance in cardiogenic shock. SMCs: smooth muscle cells; SVR: systemic vascular resistance; RAAS: renin–angiotensin–aldosterone system; CO: cardiac output.

Against this mechanistic background, the present data renew interest in thyroid hormone as a potential therapeutic agent in acute cardiac failure. The biological plausibility is compelling: triiodothyronine enhances myocardial contractility and relaxation, lowers systemic vascular resistance, and amplifies catecholamine responsiveness by increasing β-adrenergic receptor density. These effects are mediated through both genomic pathways and rapid non-genomic signaling cascades^20,37^ (Figure 7). Given the physiological derangements characteristic of cardiogenic shock: low output, systemic vasoconstriction, and adrenergic exhaustion; the cardiovascular actions of thyroid hormone are mechanistically aligned with the hemodynamic goals of acute management. At the same time, these observations should be interpreted as hypothesis-generating; any therapeutic implications require confirmation in adequately powered prospective interventional studies.

Consistent with this cautious interpretation, thyroid hormone supplementation has been explored in carefully selected patient populations within early clinical proof-of-concept studies. In a pilot investigation, patients with dilated cardiomyopathy and reduced thyroid hormone levels demonstrated improvements in cardiac output and systemic vascular resistance following short-term triiodothyronine infusion.^38^ Comparable observations were reported in postoperative cardiac surgery cohorts, in which triiodothyronine administration was associated with a reduced requirement for inotropic support.^39^

Beyond these individual studies, a broader body of experimental and early clinical evidence indicates that thyroid hormones influence key determinants of cardiovascular performance and are frequently suppressed in advanced cardiac disease. Meta-analyses in cardiac surgery populations have described improvements in cardiac index without an accompanying increase in mortality,^40^ while targeted hormone replacement in selected heart failure cohorts with low triiodothyronine has been associated with improved ventricular function.^41^ In addition, small observational reports have documented hemodynamic stabilization following intravenous thyroxine administration in patients refractory to conventional inotropic therapy.^42^

However, the application of thyroid hormone replacement in critically ill populations necessitates careful evaluation, as non-selective administration may entail adverse hemodynamic and metabolic consequences.

Potential risks associated with thyroid hormone replacement include arrhythmogenic effects, increased myocardial oxygen consumption, hypotension secondary to excessive vasodilation, and iatrogenic thyrotoxicosis.^40,43^ Although meta-analyses in patients undergoing cardiac surgery have not demonstrated a significant increase in atrial fibrillation,^42^ the transferability of these findings to cardiogenic shock is limited, given the profound hemodynamic instability and frequent vasopressor dependence characteristic of this population. Accordingly, any consideration of thyroid hormone supplementation in cardiogenic shock would require rigorous hemodynamic monitoring and careful dose titration. Current guidelines from both the American Thyroid Association and the American Heart Association support thyroid hormone replacement in selected clinical contexts, while explicitly acknowledging the need for further evidence in critically ill patients.^20,37^

Our findings suggest that dynamic alterations of the thyroid axis constitute an integral component of the systemic response to cardiogenic shock rather than a purely incidental phenomenon. Although the present study does not support therapeutic intervention, it delineates a temporally defined phase in which suppression of thyroid hormone levels coincides with pronounced clinical vulnerability. In this context, serial assessment of thyroid hormones may add incremental value to early risk stratification when interpreted alongside established hemodynamic and inflammatory markers. Future prospective studies with adequate power and temporal resolution are required to clarify whether thyroid axis suppression represents a modifiable contributor to disease progression or primarily reflects downstream severity within multisystem failure.

### Limitations

This was a single-center observational study with a limited sample size, which restricts generalizability and precludes causal inference. Although thyroid hormones were measured at standardized intervals, circadian variation and short-term fluctuations may not have been fully captured, and residual confounding cannot be excluded despite careful clinical characterization of the cohort.

The modest cohort size and limited number of events restrict statistical power for time-to-event analyses and limit the precision of hazard ratio estimates. Accordingly, effect sizes derived from Cox regression should be interpreted as exploratory measures of association rather than definitive estimates, and small-to-moderate effects may have remained undetected. Cohen’s d was used solely to describe standardized differences in continuous variables between groups and was not applied to survival analyses. Assessment of multiple thyroid hormones across repeated time points increases susceptibility to type I error. Although we used repeated-measures modeling, penalized regression, permutation testing, bootstrap resampling, and Bayesian sensitivity analyses to assess robustness and directional consistency, multiplicity and imprecision cannot be fully eliminated in a cohort of this size. Consequently, emphasis was placed on consistency of hormonal trajectories across complementary analytical approaches rather than on isolated *p*-values.

Finally, although patients receiving amiodarone or isoproterenol were excluded, the widespread use of norepinephrine may have influenced thyroid hormone dynamics through α-adrenergic effects on hormone synthesis and secretion. This should be considered when interpreting endocrine responses in the setting of acute cardiogenic shock.

## Conclusion

In conclusion, this study demonstrates dynamic alterations in thyroid hormone levels in patients with cardiogenic shock, with lower free triiodothyronine levels associated with adverse outcomes. These changes occur early and persist over time, suggesting a role of fT3 in risk stratification in this high-risk population. Given the observational design and limited sample size, these findings should be interpreted with appropriate caution. Further studies in larger cohorts are warranted to confirm their prognostic relevance and to explore potential therapeutic implications

## Supplemental Material

sj-docx-1-inc-10.1177_17511437261454174 – Supplemental material for The thyroid-heart axis-hormone dynamics and outcomes in cardiogenic shock following myocardial infarction Supplemental material, sj-docx-1-inc-10.1177_17511437261454174 for The thyroid-heart axis-hormone dynamics and outcomes in cardiogenic shock following myocardial infarction by Priyanka Boettger, Jamschid Sedighi, Laura Pallmann, Patrick Kellner, Henning Lemm, Roland Prondzinsky, Thomas Karrasch, Karl Werdan and Michael Buerke in Journal of the Intensive Care Society
