# Supplementary material for: The thyroid-heart axis-hormone dynamics and outcomes in cardiogenic shock following myocardial infarction
Source: J Intensive Care Soc. 2026 Jun 23:17511437261454174. Online ahead of print. doi: 10.1177/17511437261454174 (PMC13290748; doi:10.1177/17511437261454174)
Supplement: sj-docx-1-inc-10.1177_17511437261454174 – Supplemental material for The thyroid-heart axis-hormone dynamics and outcomes in cardiogenic shock following myocardial infarction [file sj-docx-1-inc-10.1177_17511437261454174.docx]

**Supplementary Table S1**

**Vasoactive, Inotropic, and Mechanical Circulatory Support Exposure During the First 72 Hours**

| **Treatment modality** | **Overall cohort (n = 41)** | **Survivors (n = 23)** | **Non-survivors (n = 18)** |
| --- | --- | --- | --- |
| **Vasoactive agents** |  |  |  |
| Norepinephrine, n (%) | 34 (83%) | 17 (74%) | 17 (94%) |
| Peak dose, µg/kg/min, median (IQR) | 0.32 (0.18–0.55) | 0.26 (0.15–0.40) | 0.45 (0.30–0.70) |
| Duration, hours, median (IQR) | 48 (24–72) | 36 (24–60) | 72 (48–96) |
| Epinephrine, n (%) | 9 (22%) | 3 (13%) | 6 (33%) |
| Peak dose, µg/kg/min, median (IQR) | 0.08 (0.05–0.12) | 0.06 (0.04–0.08) | 0.10 (0.07–0.14) |
| Dobutamine, n (%) | 33 (80%) | 18 (78%) | 15 (83%) |
| Peak dose, µg/kg/min, median (IQR) | 6.0 (4.0–8.0) | 5.0 (4.0–7.0) | 7.0 (5.0–9.0) |
| Levosimendan, n (%) | 18 (44%) | 11 (48%) | 7 (39%) |
| **Antiarrhythmic therapy** |  |  |  |
| Amiodarone exposure, n (%) | 15 (37%) | 7 (30%) | 8 (44%) |
| **Mechanical circulatory support (MCS)** |  |  |  |
| Any MCS, n (%) | 31 (76%) | 15 (65%) | 16 (89%) |
| IABP, n (%) | 17 (41%) | 9 (39%) | 8 (44%) |
| Impella, n (%) | 9 (22%) | 4 (17%) | 5 (28%) |
| VA-ECMO, n (%) | 7 (17%) | 2 (9%) | 5 (28%) |
| MCS duration, days, median (IQR) | 3 (2–5) | 2 (1–4) | 5 (3–7) |

***Abbreviations*** *IABP, intra-aortic balloon pump; VA-ECMO, veno-arterial extracorporeal membrane oxygenation; MCS, mechanical circulatory support; IQR, interquartile range.*

*Treatment variables are presented descriptively to contextualize clinical severity and management during cardiogenic shock. Given the observational design and limited sample size, these exposures were not interpreted as independent causal determinants of endocrine trajectories.*
